# Unterricht in Zeiten von Corona: Ein Blick auf die Herausforderungen aus der Sicht von Unterrichts- und Instruktionsforschung

**DOI:** 10.1007/s42010-020-00088-2

**Published:** 2020-10-15

**Authors:** Thamar Voss, Jörg Wittwer

**Affiliations:** 1 Institut für Erziehungswissenschaft, Empirische Schul- und Unterrichtsentwicklungsforschung, Universität Freiburg, Rempartstr. 11, 79098 Freiburg, Deutschland; 2 Institut für Erziehungswissenschaft, Empirische Lehr- und Lernforschung, Universität Freiburg, Freiburg, Deutschland

**Keywords:** Corona-Pandemie, Unterrichtsforschung, Unterrichtsqualität, Instruktionsforschung, digitales Lernen, Unterrichtsplanung, COVID-19 pandemic, Teaching research, Teaching quality, Instructional design, Digital learning, Lesson planning

## Abstract

Die Corona-Pandemie führte infolge der Schulschließungen zu einer Ausnahmesituation, die Lehrkräfte vor neue und unvorhersehbare Herausforderungen stellte. In diesem Artikel führen wir die Unterrichts- und Instruktionsforschung zusammen, um die Frage zu beantworten, wie es Lehrkräften trotz der Einschränkungen einer Pandemie gelingen kann, erfolgreichen Unterricht umzusetzen. Erstens stellen wir die Nachteile einer ausschließlichen Betrachtung der Sichtstrukturen für die Diskussion über guten Unterricht unter Pandemiebedingungen vor. Zweitens zeigen wir auf der Grundlage von Tiefenstrukturen die Herausforderungen einer Pandemie für die Gestaltung eines lernförderlichen Unterrichts im Sinne der kognitiven Aktivierung, konstruktiven Unterstützung und Effizienz des Klassenmanagements auf. Drittens konkretisieren wir anhand von Lehr-Lern-Elementen, wie Lehrkräfte auf der Grundlage von Lernzielen das Lernen von Schülerinnen und Schülern unter Pandemiebedingungen hinsichtlich Motivierung, Vermittlung, Weiterverarbeitung, Üben, Transfer und Rückmeldung fördern können. Dabei gehen wir auch auf die besondere Rolle der elterlichen Unterstützung ein. Viertens leiten wir Empfehlungen für das Unterrichten unter Pandemiebedingen ab und diskutieren, wie die Corona-Pandemie als Chance begriffen werden kann, um neue Erkenntnisse über die Gestaltung individuellen Lernens zu erhalten, die auch zukünftigen Unterricht bereichern können.

## Abstract (en)

As a consequence of the COVID-19 pandemic, the nationwide school closures caused exceptional circumstances that created new and unpredictable challenges for teachers. In this article, we synthesize research on learning and instruction to examine how teachers can teach effectively in a pandemic. First, we shed light on the disadvantages associated with exclusively taking a view on the surface structures of teaching in the discussion about good teaching under pandemic conditions. Second, on the basis of the deep structures of teaching, we present challenges caused by a pandemic for high-quality teaching in terms of cognitive activation, student support and classroom management. Third, we discuss more specifically how instructional activities can be intentionally designed by teachers with regard to stimulating motivation, presenting information, processing information, practicing, enhancing transfer, and providing feedback to facilitate learning in times of a pandemic. In this context, we also discuss the important role of parental support for learning. Fourth, we provide recommendations for good teaching under pandemic conditions and discuss how the COVID-19 pandemic can be seen as a chance for research to generate knowledge on new forms of individual learning that could enrich future teaching.

## Einführung

Aufgrund der Corona-Pandemie wurden die Schulen im März 2020 in allen deutschen Bundesländern geschlossen, eine Situation, die es in dieser Form in Deutschland noch nie gab. Schülerinnen und Schüler, Lehrkräfte sowie Eltern standen gleichermaßen vor großen Herausforderungen. Ohne Vorlauf musste während der Zeit der Schulschließung dafür Sorge getragen werden, dass den Kindern und Jugendlichen das Recht auf Bildung nicht verwehrt wird und ihnen möglichst wenig Nachteile entstehen. Eine Wiederöffnung der Schulen wurde im Rahmenkonzept des Kultusministeriums Ende April 2020 (Ständige Konferenz der Kultusminister der Länder in der Bundesrepublik Deutschland 2020) beschlossen und damit eine schrittweise Rückkehr zum Präsenzunterricht eingeleitet. In den ersten Wochen nach der Veröffentlichung des Konzepts fand in den meisten Bundesländern ein Wechsel zwischen Präsenzunterricht in kleineren Gruppen und Unterricht über die Distanz statt. Dieser Wechsel wurde in den einzelnen Bundesländern unterschiedlich ausgestaltet und dauerte in den weiterführenden Schulen in den meisten Bundesländern bis zu den Sommerferien 2020 an.

In Umfragen zur Coronazeit gab der Großteil der Lehrkräfte an, Lernmaterialien für das individuelle Lernen der Schülerinnen und Schüler im häuslichen Umfeld bereitgestellt zu haben (Forsa Politik- und Sozialforschung 2020; Vodafone Stiftung Deutschland 2020a). Jedoch hatten die Schulen kaum Zeit, sich auf die Ausnahmesituation vorzubereiten, weshalb häufig ein Gesamtkonzept fehlte (Vodafone Stiftung Deutschland 2020a). So gab es auch zwischen Lehrkräften einer Schule große Unterschiede in der Auswahl und didaktischen Gestaltung der Lernangebote, der Art der Bereitstellung von Lernmaterialien und der Wahl der Kommunikationsform, um sich mit Schülerinnen und Schülern auszutauschen (Forsa Politik- und Sozialforschung 2020; Institut für Schulentwicklungsforschung, Technische Universität Dortmund 2020; Vodafone Stiftung Deutschland 2020a; Wößmann et al. 2020). Obwohl die Lehrkräfte angaben, den Großteil der Schülerinnen und Schüler mit ihren Lernmaterialien während der Coronazeit erreicht zu haben, zweifelte die Mehrheit den Lernerfolg ihrer Schülerinnen und Schüler an (z. B. Vodafone Stiftung Deutschland 2020a). Zudem stand häufig das Halten des Lernstandes durch das Wiederholen bereits behandelter Lerninhalte im Mittelpunkt des Unterrichts (Drijvers 2020; Institut für Schulentwicklungsforschung, Technische Universität Dortmund 2020; Vodafone Stiftung Deutschland 2020a). Zugleich waren viele Eltern mit den von Lehrkräften bereitgestellten Lernangeboten unzufrieden (Huber et al. 2020) und äußerten den Wunsch nach mehr Unterstützung für das Lernen der Kinder (Vodafone Stiftung Deutschland 2020b). Wie die Ergebnisse verdeutlichen, war es offensichtlich nicht einfach, in Zeiten von Corona guten Unterricht umzusetzen.

Vor diesem Hintergrund verfolgen wir das Ziel, auf der Grundlage von Erkenntnissen aus Unterrichts- und Instruktionsforschung Hinweise zu liefern, wie erfolgreiches Unterrichten unter Pandemiebedingungen gelingen kann. Im ersten Schritt beleuchten wir aus der Perspektive der Unterrichtsforschung die Bedeutung der Unterscheidung von Sicht- und Tiefenstrukturen für die Diskussion von gutem Unterricht unter den Einschränkungen einer Pandemie und analysieren auf der Grundlage der Basisdimensionen der Unterrichtsqualität das Unterrichten in Zeiten von Corona. Im zweiten Schritt stellen wir aus der Perspektive der Instruktionsforschung vor, wie Lehr-Lern-Elemente zu gestalten sind, um Unterricht unter Pandemiebedingungen im Sinne der Basisdimensionen der Unterrichtsqualität möglichst lernwirksam umzusetzen. Im dritten Schritt leiten wir Empfehlungen ab, die für die Gestaltung individueller Lernphasen nicht nur im Fall weiterer Einschränkungen einer Pandemie nützlich sind, sondern auch generell den Unterricht bereichern können.

## Unterricht unter Pandemiebedingungen: Eine Analyse auf der Grundlage der Basisdimensionen guten Unterrichts

### Die Bedeutung der Unterscheidung von Sicht- und Tiefenstrukturen für die Diskussion von gutem Unterricht

Die deutschsprachige Unterrichtsforschung unterscheidet häufig zwischen Sicht- und Tiefenstrukturen des Unterrichts (Decristan et al. 2020; Oser und Baeriswyl 2001). *Sichtstrukturen* umfassen beobachtbare Charakteristika von Lehr-Lern-Arrangements wie die Rahmenbedingungen (z. B. Organisationsformen wie Klassenunterricht oder Förderunterricht), methodische Großformen (z. B. direkte Instruktion), Sozialformen (z. B. Partner‑, Einzel- oder Gruppenarbeit) oder das eingesetzte Medium (z. B. Computer). Bei derselben methodischen Großform, Sozialform oder bei demselben Medium können die Lehr-Lern-Interaktionen jedoch sehr unterschiedlich gestaltet sein. Beispielsweise kann eine Gruppenarbeit so umgesetzt werden, dass sich alle Schülerinnen und Schüler kognitiv mit den Lerninhalten auseinandersetzen, indem sie einen individuellen Beitrag zur Erreichung des Gruppenerfolgs leisten (Johnson und Johnson 1994). Allerdings kann eine Gruppenarbeit auch weniger lernörderlich gestaltet sein, so dass einzelne Lernende nicht aktiv sein müssen. Merkmale dieser Ebene des Unterrichts, die weniger gut direkt beobachtbar sind, werden üblicherweise als *Tiefenstrukturen* des Unterrichts bezeichnet (Kunter und Trautwein 2013). Die Tiefenstrukturen werden unterschiedlich definiert, häufig jedoch auf die Qualität der Interaktionen zwischen Lehrenden und Lernenden sowie die Qualität der Auseinandersetzung der Lernenden mit den Lerninhalten bezogen (Decristan et al. 2020). Insbesondere diese Tiefenstrukturen des Unterrichts sind bedeutsam für den Lernerfolg der Schülerinnen und Schüler (z. B. Lipowsky et al. 2009; Praetorius et al. 2018). Sie werden daher häufig zur Beurteilung der Qualität des Unterrichts herangezogen und *Basisdimensionen der Unterrichtsqualität* genannt (Decristan et al. 2020).

Die Unterscheidung zwischen Sicht- und Tiefenstrukturen ist von Bedeutung, weil sich die Diskussion, wie guter Unterricht unter Pandemiebedingungen gelingen kann, oft auf die Ebene der Sichtstrukturen beschränkt. Beispielsweise wird diskutiert, ob und wie guter Unterricht möglich ist, wenn weniger kooperative Sozialformen eingesetzt werden können, da die Schülerinnen und Schüler allein zu Hause lernen oder beim Lernen in der Schule Abstand zueinander einhalten müssen. Die Qualität des Unterrichts ist jedoch nicht primär durch die Wahl einer bestimmten Methode oder Sozialform, sondern durch deren Umsetzung in Bezug auf die Tiefenstrukturen bestimmt.

Die mangelnde Berücksichtigung der Tiefenstrukturen wird auch deutlich, wenn das Lernen in Zeiten von Corona auf die Forderung nach Digitalisierung an den Schulen reduziert wird. In Befragungen unter Lehrkräften wird der größte Verbesserungsbedarf für das individuelle Lernen im häuslichen Umfeld in Zeiten von Corona in der digitalen Kompetenz der Lehrkräfte und der Digitalisierung an den Schulen gesehen (Forsa Politik- und Sozialforschung 2020; Vodafone Stiftung Deutschland 2020a). Digitale Elemente können den Präsenzunterricht bereichern und auch unter Pandemiebedingungen ein wichtiges Hilfsmittel darstellen (Klieme 2020; Köller et al. 2020). Allerdings sind digitale Lehr-Lernmethoden ein Merkmal der Sichtstrukturen (Scheiter und Lachner 2019). Folglich ist eine digitale Unterrichtsform nicht per se besser oder schlechter als eine analoge Unterrichtsform. Zwar weist die Forschung darauf hin, dass digitale Lerntools wirksam sein können (z. B. Hillmayr et al. 2020), die Effektivität jedoch stark variiert und von ihrer konkreten Funktion abhängt (z. B. Hillmayr et al. 2017). Die Herausforderung besteht demnach darin, digitale Werkzeuge, die für das Lernen im häuslichen Umfeld unter Pandemiebedingungen angewendet werden können, lernwirksam im Sinne der Tiefenstrukturen des Unterrichts einzusetzen.

### Tiefenstrukturen: Basisdimensionen der Unterrichtsqualität

In der deutschsprachigen Unterrichtsforschung werden Merkmale der Tiefenstrukturen des Unterrichts häufig anhand von drei Basisdimensionen der Unterrichtsqualität beschrieben (z. B. Klieme 2018; Praetorius et al. 2018): *Potenzial zur kognitiven Aktivierung, konstruktive Unterstützung* und *Effizienz der Klassenführung*. Diese Basisdimensionen wurden empirisch aus einer Vielzahl an Skalen zur Messung der Unterrichtsqualität gewonnen (z. B. Klieme et al. 2001). Sie werden zur Beurteilung der Qualität von Unterricht in verschiedenen Lernkontexten (z. B. Fach, Jahrgangsstufe; siehe Praetorius et al. 2020) und Sichtstrukturen (z. B. methodische Großform, Sozialform; siehe Kunter und Trautwein 2013) herangezogen. Die Bedeutung der drei Dimensionen für die kognitive und motivational-emotionale Entwicklung von Schülerinnen und Schülern wurde in zahlreichen Studien aufgezeigt (z. B. Baumert et al. 2010; Fauth et al. 2019).

#### Potenzial zur kognitiven Aktivierung

Das Potenzial zur kognitiven Aktivierung bezieht sich auf die Anregung des Unterrichts zu Denkprozessen auf hohem kognitiven Niveau (Leuders und Holzäpfel 2011). Demnach hat ein Unterricht ein hohes Potenzial zur kognitiven Aktivierung, wenn er auf Verstehen ausgerichtet ist und zur kognitiven Auseinandersetzung auf der Basis des Vorwissens der Schülerinnen und Schüler anregt (Klieme 2018).

Auch für das Lernen unter Pandemiebedingungen ist es wichtig, dass sich Schülerinnen und Schüler vertieft mit Lerninhalten auseinandersetzen (Klieme 2020). Lehrkräfte stellten ihren Schülerinnen und Schülern während der Schulschließungen in der Regel Lernmaterialien für das Lernen im häuslichen Umfeld bereit (Forsa Politik- und Sozialforschung 2020; Vodafone Stiftung Deutschland 2020a). Dabei wurden am häufigsten Arbeitsblätter verwendet. Auch Erklärvideos kamen zum Einsatz (Forsa Politik- und Sozialforschung 2020; Vodafone Stiftung Deutschland 2020a; Wößmann et al. 2020). Synchroner Online-Unterricht über Konferenzsysteme fand dagegen deutlich seltener statt. Ebenso wurden digitale Lernprogramme kaum verwendet (Forsa Politik- und Sozialforschung 2020; Vodafone Stiftung Deutschland 2020a; Wößmann et al. 2020). Dass Schülerinnen und Schüler in der Zeit der Schulschließungen nicht immer angemessen zu einer kognitiven Aktivierung angeregt wurden, legen Äußerungen von befragten Eltern nahe. Danach stellten Lehrkräfte zu viele Lernmaterialien ohne klare Fokussierung bereit. Zudem stand häufig das bloße Abarbeiten von Arbeitsblättern im Vordergrund (Huber et al. 2020).

Ein hohes Potenzial zur kognitiven Aktivierung unter Pandemiebedingungen kann beispielsweise durch Aufgaben zur gezielten Aktivierung des Vorwissens oder durch die Anreicherung von Arbeitsblättern mit Aufforderungen, Lösungsansätze zu vergleichen, erreicht werden. Auch kooperative Lernformen (z. B. Online-Diskussionen) können die kognitive Auseinandersetzung der Schülerinnen und Schüler mit Lerninhalten fördern, indem die Schülerinnen und Schüler zur gegenseitigen Erklärung angeregt werden.

#### Konstruktive Unterstützung

Die konstruktive Unterstützung umfasst sowohl strukturierende als auch sozio-emotionale Aspekte (Kunter und Voss 2013). Strukturierende Aspekte zielen darauf ab, Schülerinnen und Schüler in die Lage zu versetzten, Aufgaben zu bewältigen, die sie ohne diese Hilfestellung noch nicht bewältigen könnten (Pea 2004), und die Lernprozesse durch konstruktives Feedback zu begleiten (Hattie und Timperley 2007). Im Hinblick auf die sozio-emotionalen Aspekte einer konstruktiven Unterstützung sollte der Unterricht so gestaltet sein, dass sich Schülerinnen und Schüler wertgeschätzt fühlen, sie als autonome und kompetente Personen geachtet werden und ein respektvoller Umgangston herrscht (Kunter und Trautwein 2013). Die Forschung zeigt, dass die konstruktive Unterstützung insbesondere für die Motivation der Schülerinnen und Schüler bedeutsam ist (Cornelius-White 2007; Kunter et al. 2013; Roorda et al. 2011).

Eine konstruktive Unterstützung ist auch für das Lernen unter Pandemiebedingungen mit den ausgedehnten Phasen individuellen Lernens und des *social distancing* wichtig (Klieme 2020). In Befragungen zum Lernen während der Schulschließungen in der Coronazeit wurde die unzureichende Begleitung der Lernprozesse durch die Lehrkräfte in Form von Rückmeldungen bemängelt (Forsa Politik- und Sozialforschung 2020; Wößmann et al. 2020). Zudem wurde das Absinken der Motivation (Forsa Politik- und Sozialforschung 2020) als eine der Hauptbefürchtungen von Lehrkräften und Eltern (Wildemann und Hosenfeld 2020) formuliert.

Eine hohe konstruktive Unterstützung kann unter Pandemiebedingungen beispielsweise durch Lernmaterialien, in denen über die Lernziele und das bevorstehende Lernen informiert wird und die zu bearbeitenden Aufgaben in überschaubare Einzelschritte zerlegt sind, erreicht werden. Zudem kann ein regelmäßiger Austausch mit den Schülerinnen und Schülern über Chats oder Konferenzsysteme zu einer wertschätzenden Begleitung das Lernens durch die Lehrkräfte beitragen.

#### Effizienz des Klassenmanagements

Die Effizienz des Klassenmanagements umfasst Strategien zur Sicherstellung eines störungsarmen Unterrichts, so dass die Schülerinnen und Schüler die Lernzeit optimal nutzen (Doyle 2006). Um dies zu erreichen, ist die Prävention von Störungen und Ablenkungen (Kounin 2006) durch klare Strukturen und ein Regelsystem notwendig (Emmer et al. 2003). Metaanalysen (z. B. Marzano 2009; Seidel und Shavelson 2007) zeigen, dass ein effizientes Klassenmanagement eine wichtige Voraussetzung für den Lernerfolg der Schülerinnen und Schüler darstellt.

Für das Lernen unter Pandemiebedingungen, insbesondere in der Zeit einer kompletten Schulschließung, spielt Klassenmanagement im Hinblick auf die Koordination des komplexen sozialen Gefüges im Klassenraum eine untergeordnete Rolle. Ein gutes Management der Lernaktivitäten der Schülerinnen und Schüler zur Maximierung der *time on task* ist jedoch gerade für das individuelle Lernen im häuslichen Umfeld zentral (Klieme 2020). Allerdings äußerte eine große Mehrheit der befragten Lehrkräfte in zwei bundesweiten Studien (Institut für Schulentwicklungsforschung, Technische Universität Dortmund 2020; Vodafone Stiftung Deutschland 2020a), dass Schülerinnen und Schüler in Zeiten von Corona nicht so effektiv wie gewöhnlich lernen konnten. Diesen Eindruck teilten die befragten Eltern, die angaben, dass ihre Kinder weniger Zeit mit dem Lernen als sonst verbrachten (Wößmann et al. 2020). Auch waren viele Schülerinnen und Schüler der Ansicht, weniger als im regulären Unterricht gelernt zu haben (Huber et al. 2020).

Die Lehrkräfte können unter Pandemiebedingungen durch eine gute Planung des Lernens zu einer hohen *time on task* beitragen. Klieme (2020) schlägt gut strukturierte Tages- und Wochenpläne vor. Diese sollten alle notwendigen Informationen enthalten, die die Schülerinnen und Schüler benötigen, um erfolgreich allein lernen zu können.

Zusammenfassend lässt sich festhalten, dass mit dem Modell der drei Basisdimensionen der Unterrichtsqualität die Besonderheiten des Lernens unter Pandemiebedingungen systematisch beleuchtet und – wie exemplarisch aufgezeigt – Ansatzpunkte zur Verbesserung des Unterrichtens in dieser besonderen Situation abgeleitet werden können. Die Basisdimensionen beschreiben die Qualität der Lehr-Lern-Situationen, die in Angebot-Nutzungs-Modellen (Überblick bei Vieluf et al. 2020) häufig als Angebotsstrukturen bezeichnet werden. Die Zusammenhänge von Merkmalen der Angebotsstruktur mit dem Lernerfolg der Schülerinnen und Schüler wurde vielfach geprüft (z. B. Übersicht bei Praetorius et al. 2018). Es wird in der Unterrichtsforschung jedoch selten untersucht, welche Lernprozesse ablaufen müssen und wie diese Lernprozesse gezielt angestoßen werden können, damit Unterricht erfolgreich ist. Vor diesem Hintergrund kann die Instruktionsforschung, in deren Mittelpunkt die Gestaltung der Lehr-Lern-Prozesse steht (Klauer und Leutner 2012), einen wesentlichen Beitrag zum Verständnis guten Unterrichts unter Pandemiebedingungen leisten.

## Guter Unterricht unter Pandemiebedingungen: Erkenntnisse aus der Instruktionsforschung

Welche Prozesse bei Lernenden anzustoßen sind, damit Unterricht im Sinne der Basisdimensionen der Unterrichtsqualität (Praetorius et al. 2018) möglichst lernwirksam ist, lässt sich mit Hilfe instruktionspsychologischer Modelle konkretisieren (z. B. Klauer und Leutner 2012; Koedinger et al. 2012; Smith und Ragan 2005). Ausgehend von einer kognitivistischen Auffassung von Lernen als ein Prozess der Informationsverarbeitung geben instruktionspsychologische Modelle an, welche Lernprozesse ablaufen müssen, damit Lehren effektiv ist. Angeregt werden die Lernprozesse durch Lehr-Lern-Elemente wie Motivieren oder Informieren, die als *instructional events *(Gagné 1965), *Lehrfunktionen* (Klauer und Leutner 2012) oder *first principles of instruction* (Merrill 2009) bezeichnet werden. Hinweise, wie die Lehr-Lern-Elemente möglichst lernwirksam zu gestalten sind, liefern Untersuchungen, in denen spezifische Merkmale einzelner Lehr-Lern-Elemente hinsichtlich ihrer Lernwirksamkeit überprüft werden (z. B. Anzahl von Beispielen in Erklärungen; Rawson et al. 2015).

Übertragen auf das Lernen unter Pandemiebedingungen stellt sich die Frage, wie die Lehr-Lern-Elemente umzusetzen sind, damit Unterricht gelingen kann. Das Lernen unter den Einschränkungen einer Pandemie, insbesondere wenn es auf Distanz im häuslichen Umfeld erfolgt, erfordert von Schülerinnen und Schülern in besonderer Weise, ihr Lernen selbst zu initiieren, geeignete Strategien zum Lernen auszuwählen und ihr Lernen eigenständig zu überwachen. Nicht alle Schülerinnen und Schüler verfügen über diese Selbstregulationsfähigkeiten. Um dennoch einen hohen Lernerfolg zu ermöglichen, ist es wichtig, die einzelnen Lehr-Lern-Elemente so zu gestalten, dass Schülerinnen und Schüler dabei unterstützt werden, eine aktive Rolle für ihr Lernen übernehmen zu können (Fischer et al. 2020). Zudem ist es Lehrkräften im Vergleich zum herkömmlichen Unterricht unter Pandemiebedingungen nur eingeschränkt möglich, unmittelbar didaktische Entscheidungen zu treffen, wenn die Lernsituation es erforderlich macht. Aus diesen Gründen ist eine besonders sorgfältige Planung des Unterrichts notwendig.

Um darzustellen, wie das Unterrichten unter den Einschränkungen einer Pandemie gestaltet werden kann, behandeln wir ausgehend von einer auf (1) Lernzielen abgestimmten Planung des Unterrichts als Lehr-Lern-Elemente (2) Motivierung, (3) Vermittlung, (4) Weiterverarbeitung, (5) Üben, (6) Transfer und (7) Rückmeldung. Zudem gehen wir aufgrund ihrer besonderen Bedeutung für das Lernen der Schülerinnen und Schüler im häuslichen Umfeld in den Zeiten einer Pandemie auf die (8) elterliche Unterstützung ein.

### Wie kann das Lernen durch die Festlegung von Lernzielen unterstützt werden?

Trotz der Einschränkungen, die sich für das Lernen unter Pandemiebedingungen ergeben, ist es wichtig, dass sich Schülerinnen und Schüler mit neuen Lerninhalten befassen und nicht nur Gelerntes wiederholen (Klieme 2020). Um die eigenständige Aneignung neuer Lerninhalte auch im häuslichen Umfeld zu unterstützen, sollten Lehrkräfte wissen, welche spezifischen Anforderungen die neuen Lerninhalte an ihre Schülerinnen und Schüler stellen. Hierzu empfiehlt sich die Festlegung von Lernzielen, die vorgeben, was Schülerinnen und Schüler nach dem Lernen können sollen (Wittwer et al. 2019). Dadurch wird geklärt, was Schülerinnen und Schüler im Lernprozess tun müssen, um ein Lernziel zu erreichen. Um ein möglichst genaues Bild vom Lernprozess zu erhalten, sollten Lehrkräfte analysieren, welche einzelnen Schritte auf dem Weg zu einem Lernziel durchzuführen sind und welches Wissen für die Durchführung jedes dieser Schritte notwendig ist (Tofel-Grehl und Feldon 2013). Dabei können Lernzieltaxonomien verwendet werden, um die Art des erforderlichen Wissens genauer zu bestimmen (z. B. Smith und Ragan 2005). Häufig unterscheiden Lernzieltaxonomien vier Wissensarten:

1. Wissen über Fakten bezieht sich auf Informationen, die man aus dem Gedächtnis abrufen kann (z. B. Definition von Bruch).
2. Mit Wissen über Konzepte können Dinge klassifiziert werden (z. B. Erkennen, was Zähler und Nenner beim Bruch sind).
3. Wissen über Prinzipien erlaubt es, Erklärungen zu geben und Vorhersagen zu treffen (z. B. erklären, ob ein Bruch größer oder kleiner wird, wenn die Zahl im Nenner größer wird).
4. Mit Wissen über Prozeduren können Handlungen durchgeführt werden, um ein bestimmtes Ziel zu erreichen (z. B. Bruch kürzen).

Die Analyse von Lernzielen ermöglicht es Lehrkräften, ein umfassendes Bild über die unterschiedlichen Anforderungen neuer Lerninhalte und die vielfältigen Arten des zu erwerbenden Wissens zu erhalten. Dadurch sollte es Lehrkräften leichter fallen, alle relevanten Informationen, die Schülerinnen und Schüler benötigen, um ein Lernziel zu erreichen, im Lernprozess bereitzustellen (Tofel-Grehl und Feldon 2013). Gerade unter Pandemiebedingungen ist es wichtig, dass Lernmaterialien vollständig sind und keine Informationen auslassen, die sich Schülerinnen und Schüler ohne direkte Unterstützung einer Lehrkraft nicht selbständig erschließen können.

Die Analyse der Lernziele ist jedoch nicht nur für die Feststellung der Anforderungen neuer Lerninhalte, sondern auch für die weitere Unterrichtsplanung nützlich. Haben Lehrkräfte die zu erreichenden Lernziele festgelegt, können sie die Gestaltung der einzelnen Lehr-Lern-Elemente auf die Lernziele abstimmen (Klauer und Leutner 2012). Beispielsweise können Lehrkräfte Lernformen auswählen, mit denen zum Lernziel passende Lernprozesse angeregt werden, und Aufgaben erstellen, bei denen das für die Erreichung eines Lernziels erforderliche Wissen anzuwenden ist. Auf diese Weise können Lehrkräfte das Unterrichten der Schülerinnen und Schüler systematisch vorbereiten, was vor allem für das eigenständige Lernen unter Pandemiebedingungen wichtig ist.

### Wie kann die Motivation der Schülerinnen und Schüler gefördert werden?

In den langen individuellen Lernphasen unter Pandemiebedingungen kann das Lernen zu Hause unter mangelnder Motivation leiden. Ergebnisse aus Befragungen weisen darauf hin, dass sowohl Lehrkräfte (Forsa Politik- und Sozialforschung 2020) als auch Eltern (Wildemann und Hosenfeld 2020) bei vielen Schülerinnen und Schülern in Zeiten der coronabedingten Schulschließungen eine geringe Motivation beobachteten.

Eine hohe Motivation der Schülerinnen und Schüler ist jedoch wichtig für eine aktive Auseinandersetzung mit Lerninhalten (z. B. Schiefele und Schaffner 2015). Nach der Erwartungs-Wert-Theorie (z. B. Wigfield und Eccles 2000) hängt die Bereitschaft, sich mit Lerninhalten zu beschäftigen, von zwei Faktoren ab, (1) dem Wert, den neue Lerninhalte für Schülerinnen und Schüler besitzen, und (2) der Erwartung, dass Schülerinnen und Schüler in der Lage sind, sich die Lerninhalte anzueignen. Beide Faktoren sollten gegenüber Schülerinnen und Schülern vor dem eigentlichen Lernen ausdrücklich angesprochen werden (Hattie und Donoghue 2016).

Um Schülerinnen und Schülern den Wert von Lerninhalten klarzumachen, ist es sinnvoll, ihnen (1) Lernziele vorzugeben (Smith und Ragan 2005). Dabei können Lehrkräfte die Wichtigkeit der Lernziele betonen, indem sie deren Bedeutung für die Lebenswelt der Schülerinnen und Schüler verdeutlichen. In Schulen findet häufig ein kumulativer Kompetenzerwerb statt. Deshalb kann Schülerinnen und Schülern auch klargemacht werden, wie wichtig die Erreichung von Lernzielen für die erfolgreiche Aneignung komplexeren Wissens ist. Zudem kann durch die (2) Auswahl von Aufgaben mit einem direkten Bezug zur Lebenswelt der Schülerinnen und Schüler der Wert der Lerninhalte verdeutlichet werden. Eine Möglichkeit, sich aktiv mit dem Nutzen von Lerninhalten auseinanderzusetzen, sind kurze (3) Nützlichkeitsinterventionen, die an den Wertvorstellungen von Schülerinnen und Schülern ansetzen. Zum Beispiel sollen die Schülerinnen und Schüler in solchen Interventionen Zitate zum Nutzen eines Faches für den Alltag evaluieren oder sie werden aufgefordert, den persönlichen Nutzen eines Faches zu reflektieren (Gaspard et al. 2015; Hulleman und Harackiewicz 2009). Diese Interventionen erweisen sich trotz der geringen Dauer als effektiv (Lazowski und Hulleman 2016). Unter Pandemiebedingungen könnten Lehrkräfte Arbeitsblätter so gestalten, dass Schülerinnen und Schüler Informationen über Lernziele erhalten, der Bezug zum eigenen Leben und Lernen hergestellt wird und sie darüber reflektieren müssen, welchen Nutzen die Lerninhalte für sie besitzen. So können Lehrkräfte auch über die Distanz zu einer Förderung der Motivation für die Auseinandersetzung mit Lerninhalten beitragen.

Um die Erwartung von Schülerinnen und Schülern beim Lernen zu unterstützen, Wissen über neue Lerninhalte erfolgreich erwerben zu können, empfiehlt es sich, sie über das bevorstehende Lernen zu informieren. Im Sinne der konstruktiven Unterstützung können Lehrkräfte hierzu unter Pandemiebedingungen in den Lernmaterialien ihren Schülerinnen und Schülern erläutern, wie sie sich durch die eingesetzten Lernformen neues Wissen aneignen, wie sie durch die Verwendung von Lernstrategien ihr Lernen selbst aktiv steuern können und wie sich ihr Wissenserwerb im Verlauf des Lernens entwickelt (Pintrich und Schunk 1996).

Bauen die Schülerinnen und Schüler durch solche Maßnahmen vor dem Lernen realistische Erwartungen auf, führt dies beim Lernen häufiger zum Erleben von Kompetenz. Nach der Selbstbestimmungstheorie (Deci und Ryan 1985) ist Kompetenzerleben ein wichtiger Aspekt, der zur Motivation der Schülerinnen und Schüler beiträgt. Auch die Anpassung der Schwierigkeit der Lernmaterialien, die Bereitstellung von Hilfsmaterialien oder regelmäßiges Feedback können bei Schülerinnen und Schülern das Erleben von Kompetenz unterstützen (z. B. Schiefele 2004). Neben dem Kompetenzerleben kann die Motivation der Schülerinnen und Schüler durch das Erleben sozialer Eingebundenheit gefördert werden (Deci und Ryan 1985). Hierzu kann unter Pandemiebedingungen die Umsetzung kooperativer Lernelemente über digitale Medien (z. B. Chat oder Videokonferenzprogramme) beitragen. Viele Videokonferenzprogramme bieten die Möglichkeit, Kleingruppen zu bilden und Formen kooperativen Lernens, in denen die Gruppenmitglieder beim Erreichen der vorgegebenen Ziele wechselseitig voneinander abhängig sind und individuell zum Erreichen des Gruppenziels beitragen müssen, umzusetzen (z. B. Gruppenpuzzle, Aronson et al. 1978; Gruppenralley, Slavin 1994). Forschungsbefunde weisen auf das Potenzial des computergestützten kooperativen Lernens für die Motivation und den Kompetenzerwerb der Schülerinnen und Schüler hin (z. B. Chen et al. 2018; Jeong et al. 2019).

### Wie kann die Vermittlung durch lernförderliche Erklärungen umgesetzt werden?

Ein großer Teil des Lernens im häuslichen Umfeld von Schülerinnen und Schülern in Zeiten von Corona bestand in der Bearbeitung von Arbeitsblättern (z. B. Wößmann et al. 2020), die üblicherweise auch Erklärungen enthalten. Die Erklärungen können von Lehrkräften selbst erstellt sein oder aus Lehrbüchern stammen und als Text (mit Bildern) oder Video präsentiert werden (Forsa Politik- und Sozialforschung 2020).

Damit Schülerinnen und Schüler unter den Einschränkungen von Pandemien die in den Erklärungen dargestellten Inhalte möglichst gut verstehen (Wittwer und Renkl 2008), ist es im Sinne der kognitiven Aktivierung wichtig, die Anwendung des zu erwerbenden Wissens in Erklärungen zu demonstrieren (Merrill 2009). Hierzu sollte die Gestaltung der Informationen und Beispiele in den Erklärungen auf das Lernziel abgestimmt werden. So kann bei Konzepten eine Definition zusammen mit Beispielen für das zu lernende Konzept gegeben werden (Tennyson und Cocchiarella 1986). Geht es um Prozeduren, sind die erforderlichen Schritte und Beispielsituationen, in denen die Schritte durchgeführt werden können, darzustellen (Renkl 2014).

In vielen Fällen zielt das Lernen auf den Erwerb von Fertigkeiten ab, die in unterschiedlichen Situationen anwendbar sind. Deshalb sollten Erklärungen auch dazu beitragen, dass Schülerinnen und Schüler von den konkreten Beispielen losgelöste und abstrahierte Wissensschemata aufbauen (Wittwer und Renkl 2008). Hierzu empfiehlt es sich, mehrere Beispiele in einer Erklärung zu präsentieren. So zeigten Rawson et al. (2015), dass der Erwerb von Wissen über Konzepte gefördert wird, wenn Lernende mehrere Beispiele, die die zu erlernenden Konzepten illustrierten, erhielten. Die ausschließliche Beschäftigung mit den Definitionen von Konzepten reichte hingegen nicht aus, um das Lernen effektiv zu unterstützen. In ähnlicher Weise ist es für den erfolgreichen Erwerb von Wissen über Prozeduren entscheidend, dass Lernende die Möglichkeit erhalten, nicht nur ein Lösungsbeispiel, sondern mehrere Lösungsbeispiele, anhand derer sie die Schritte einer Prozedur nachvollziehen können, zu studieren (Sweller und Cooper 1985).

Die Empfehlung, mehrere Beispiele in Erklärungen zu präsentieren, verdeutlicht, dass Schülerinnen und Schüler nicht sofort ihr neu erworbenes Wissen anwenden sollten. So fanden Zamary und Rawson (2018), dass Lernende, die nach einer Einführung von Konzepten Beispiele zu diesen Konzepten generieren sollten, im Vergleich zu Lernenden, die nach einer Einführung von Konzepten Beispiele zu diesen Konzepten erhielten, einen geringeren Lernerfolg aufwiesen. Auch wenn es um den Erwerb von Wissen über Prozeduren geht, weist die Forschung darauf hin, dass Lernende nicht zu früh eigenständig Aufgaben bearbeiten sollten. Stattdessen ist es lernförderlicher, zunächst Lösungsbeispiele zu studieren (Renkl 2014). Deshalb sollten Arbeitsblätter, die für das Unterrichten unter Pandemiebedingungen eingesetzt werden, so gestaltet sein, dass der Übergang von Erklärungen zu Übungsaufgaben nicht zu früh erfolgt.

In den Zeiten einer Pandemie bietet es sich an, Erklärungen den Schülerinnen und Schülern über das Internet bereitzustellen. Dabei können Lehrkräfte digitale Werkzeuge einsetzen, um Erklärungen multimedial zu gestalten. Allerdings ist es nicht ratsam, Erklärungen per se multimedial zu präsentieren. Stattdessen ist im Einzelfall zu entscheiden, ob eine multimediale Gestaltung von Erklärungen zur Erreichung eines Lernziels beiträgt. Beispielsweise sind multimediale Erklärungen im Sinne des *Multimediaprinzips* von Mayer (2014) nur dann effektiv, wenn ein Bild zusätzlich zu einem Text lernrelevante Informationen vermittelt oder die in einem Text dargestellten Informationen verständlicher macht (Ainsworth 2006). Damit multimedial präsentierte Erklärungen das Lernen unterstützen, ist es auch notwendig, dass Schülerinnen und Schüler die multimedialen Elemente einer Erklärung miteinander verknüpfen können (Renkl und Scheiter 2017). Um diesen Prozess zu unterstützen, empfiehlt es sich nach Mayer (2014), unterschiedliche Gestaltungsprinzipien umzusetzen. Hierzu gehört es beispielsweise, sprachliche und bildliche Informationen in unterschiedlichen Sinnesmodalitäten darzubieten (*Modalitätsprinzip*), in räumlicher Nähe anzuordnen (*räumliches Kontiguitätsprinzip*) und simultan zu präsentieren (*zeitliches Kontiguitätsprinzip*).

Eine besondere Form multimedialer Erklärungen sind Erklärvideos, die auch in Zeiten von Corona eingesetzt wurden (Forsa Politik- und Sozialforschung 2020). Üblicherweise werden in Erkärvideos sprachliche Informationen mündlich vorgetragen und mit bewegten Bildern kombiniert. Auch wenn Erklärvideos im Sinne des Multimediaprinzips (Mayer 2014) das Lernen unterstützen können, ergeben sich bei ihrer Gestaltung nach Schmidt-Borcherding (2020) doch spezifische Herausforderungen: Aufgrund ihres dynamischen Charakters sind die Informationen in Erklärvideos flüchtig und damit der Aufmerksamkeit von Schülerinnen und Schülern nicht lange zugänglich. Deshalb ist es wichtig, dass die Verarbeitung der Informationen in Erklärvideos durch Schülerinnen und Schüler kontrolliert werden kann (z. B. durch Stoppen des Erklärvideos). Zudem erfordert die Auseinandersetzung mit den Informationen in Erklärvideos andere Strategien (z. B. Annotieren) als solche, die Schülerinnen und Schüler beim Lesen von Texten (z. B. Unterstreichen) bereits gewohnt sind. Aufgrund dieser Herausforderungen sollten Lehrkräfte bei der Vermittlung von Lerninhalten abwägen, ob der erwartete Mehrwert von Erklärvideos den erhöhten Aufwand für ihre Erstellung rechtfertigt.

### Wie kann die Weiterverarbeitung von Lerninhalten gezielt gefördert werden?

Findet das Lernen unter Pandemiebedingungen vor allem im häuslichen Umfeld statt, besteht aufgrund der fehlenden Begleitung durch die Lehrkraft die Gefahr, dass sich Schülerinnen und Schüler mit neuen Lerninhalten nur oberflächlich beschäftigen. Trotzdem können sie in diesem Fall irrtümlicherweise annehmen, viel gelernt zu haben, was sich negativ auf das weitere Lernen auswirken kann (z. B. Prinz et al. 2019). Beispielsweise könnten Schülerinnen und Schüler eine Erklärung lesen und unter dem Eindruck, die Erklärung verstanden zu haben, keine weiteren Lernaktivitäten unternehmen. Um dieser Gefahr vorzubeugen, ist es im Sinne der kognitiven Aktivierung empfehlenswert, dass die bereitgestellten Lernmaterialien eine aktive Auseinandersetzung mit neuen Lerninhalten gezielt anregen. Dadurch können Schülerinnen und Schüler nicht nur verstehen, wie die Informationen im Lernstoff zusammenhängen, sondern auch die neuen Lerninhalte mit ihrem bereits vorhandenen Wissen verknüpfen (Klauer und Leutner 2012).

Eine aktive Auseinandersetzung mit neuen Lerninhalten kann durch generative Aktivitäten wie *Visualisieren, Selbsterklären *und *gegenseitiges Erklären* angestoßen werden (Fiorella und Mayer 2016). Um eine hohe Lernwirksamkeit zu erreichen, empfiehlt es sich, die generativen Aktivitäten vor dem Hintergrund der zu erreichenden Lernziele zu gestalten. Dabei können generative Aktivitäten miteinander kombiniert werden (Fiorella und Kuhlmann 2020). Schülerinnen und Schüler können generative Aktivitäten auch mit Hilfe digitaler Werkzeuge umsetzen (z. B. Tablets für Visualisierungen) und die dabei erstellten Produkte ihren Lehrkräften zu Verfügung stellen. Dadurch können sich Lehrkräfte unter Pandemiebedingungen ein genaueres Bild von den Lernprozessen der Schülerinnen und Schüler verschaffen.

Visualisierungen sind dazu geeignet, die Organisation von Informationen im Lernstoff zu unterstützen (McCrudden und Rapp 2017). Beispielsweise kann nach dem Lesen einer Erklärung zu einer Prozedur ein Sequenzdiagramm erstellt werden, um die dargestellten Schritte einer Prozedur in ihrer zeitlichen Abfolge zu visualisieren. Für das vertiefte Verstehen eines Konzepts ist es möglich, realistische Abbildungen anzufertigen, die eine hohe Ähnlichkeit mit dem dargestellten Sachverhalt besitzen (z. B. realitätsnahe Darstellung des menschlichen Herzens).

Selbsterklärungen dienen dazu, sich den Sinn von neuen Informationen im Lernstoff zu erschließen. Dies kann dadurch geschehen, dass Schülerinnen und Schüler Verknüpfungen zwischen Informationen im Lernstoff herstellen und Schlussfolgerungen ziehen (Fonseca und Chi 2011). Beispielsweise können Schülerinnen und Schüler durch Prompts aufgefordert werden, verschiedene Beispiele zu einem Konzept miteinander zu vergleichen und sich selbst erklären, welche Gemeinsamkeiten und Unterschiede zwischen den Beispielen bestehen (Alfieri et al. 2013). Beim Erlernen einer Prozedur können sich Selbsterklärungen darauf beziehen, wie sich die einzelnen Schritte in einem Lösungsbeispiel auf ein zugrunde liegendes Prinzip (z. B. mathematisches Theorem) beziehen (Renkl 2014).

Schülerinnen und Schüler können nicht nur sich selbst, sondern in der Rolle von Lehrenden auch ihren Mitschülerinnen und Mitschülern Erklärungen geben. Das gegenseitige Erklären erfordert es, die zu vermittelnden Lerninhalte aufzubereiten und mit dem eigenen Wissen zu verknüpfen (Fiorella und Mayer 2013). Unter Pandemiebedingungen empfiehlt es sich, dass sich Schülerinnen und Schüler über Videokonferenzprogramme gegenseitig Erklärungen geben oder sie mit digitalen Werkzeugen Erklärvideos erstellen. Hoogerheide et al. (2019) zeigen, dass eigenständig generierte Erklärvideos nicht nur lernwirksam sind, sondern auch motivierend wirken können.

Generative Aktivitäten können auch dazu beitragen, den Lernerfolg indirekt zu unterstützen, indem sie Schülerinnen und Schülern dabei helfen, ihr eigenes Verstehen im Lernprozess genauer einzuschätzen. Ergebnisse aus der Metaanalyse von Prinz et al. (2020) zeigen, dass Lernende durch die Vervollständigung von Diagrammen akkurater beurteilten, wie gut sie die Inhalte von Texten verstehen. Besonders wenn das Lernen unter Pandemiebedingungen allein stattfindet, ist eine genaue Überwachung der eigenen Verstehensprozesse wichtig, um das Lernen optimal zu steuern (Fischer et al. 2020).

### Wie kann der Lernerfolg durch Üben unterstützt werden?

Arbeitsblätter, die in Zeiten von Corona häufig eingesetzt wurden (z. B. Wößmann et al. 2020), enthielten zusätzlich zu Erklärungen häufig Übungsaufgaben. Damit sollen Schülerinnen und Schüler die Gelegenheit erhalten, ihr neues Wissen anzuwenden. Im Sinne eines formativen Assessment (Herppich et al. 2014) dient das Üben vorrangig dem weiteren Lernen (*assessment for learning*) und nicht bereits der Überprüfung des erworbenen Wissens (*assessment of learning*). Um das weitere Lernen optimal zu unterstützen, ist es wichtig, dass Lehrkräfte die Übungsaufgaben auf die Lernziele abstimmen. Auf diese Weise können einzelne Aspekte des für die Lernzielerreichung notwendigen Wissens systematisch eingeübt werden. Geht es um Konzepte, könnten die Übungsaufgaben es erfordern, vorgegebene Beispiele zu einem Konzept zu erkennen, selbst Beispiele für ein Konzept zu geben oder die Merkmale, die ein Konzept auszeichnen, zu erklären. Bei Übungsaufgaben zu Prozeduren könnte es nötig sein, die Schritte einer Prozedur durchzuführen, die einzelnen Schritte zu nennen oder die Ausführung einer Prozedur zu beurteilen.

Unter Pandemiebedingungen empfiehlt es sich besonders, das Potenzial, das in der Gestaltung von Übungsaufgaben für das Lernen liegt, auszuschöpfen. Hinsichtlich der Art des Übens zeigt die Forschung zum *testing effect* (Adesope et al. 2017), dass es lernförderlicher ist, Schülerinnen und Schüler ihr Wissen über neue Lerninhalte aktiv anwenden zu lassen (z. B. durch Beantwortung von Fragen) und sie nicht lediglich zur erneuten Verarbeitung der Lerninhalte (z. B. durch Lesen) aufzufordern. Was die zeitliche Umsetzung des Übens betrifft, ist verteiltes Üben für den langfristigen Lernerfolg gewöhnlich besser als massiertes Üben (z. B. Dunlosky et al. 2013). Deshalb sollten Schülerinnen und Schüler durch die Aufbereitung der Lernmaterialien dazu angeregt werden, das Lernen auf mehrere kürzere Phasen zu verteilen anstatt eine lange Lernphase einzuplanen. Es stellt sich auch die Frage, in welcher Reihenfolge Schülerinnen und Schüler Übungsaufgaben bearbeiten sollten. Rohrer et al. (2015) zeigten, dass es lernförderlicher ist, Übungsaufgaben, die jeweils unterschiedliches Wissen und nicht dasselbe Wissen erfordern, nacheinander bearbeiten zu lassen. So eine Reihenfolge verlangt von Schülerinnen und Schülern, auf der Grundlage der jeweiligen Übungsaufgabe zu entscheiden, welches Wissen für die Lösung der Übungsaufgabe notwendig ist. Dadurch wird verhindert, dass bereits vor der Bearbeitung einer Übungsaufgabe klar ist, welches Wissen anzuwenden ist, wodurch ein echtes Verstehen der Anwendungsbedingungen des Wissens ausbleiben würde.

### Wie kann der Transfer von Wissen gefördert werden?

Aus den Befragungen, die zeigen, dass Schülerinnen und Schüler in Zeiten von Corona vor allem Lerninhalte wiederholten (z. B. Drijvers 2020), wird nicht ersichtlich, ob sie dabei auch aufgefordert wurden, ihr erworbenes Wissen auf die eigene Lebenswelt zu übertragen. Für ein vertieftes Verständnis von Lerninhalten ist es jedoch wichtig, neues Wissen zu nutzen, um es in authentischen Lebenssituationen anzuwenden (Klauer 2011).

Zur Förderung des Transfers ergeben sich grundsätzlich zwei Möglichkeiten: Erstens können Lehrkräfte Aufgaben vorgeben, in denen Schülerinnen und Schüler ihr neues Wissen auf persönlich relevante Lebenssituation übertragen müssen. Beispielsweise stellen Bohl et al. (2015) dar, dass Aufgaben danach unterschieden werden können, wie neuartig eine Situation ist, in der Wissen anzuwenden ist, und wie stark der Bezug zur eignen Lebenswelt ist. Folglich sollten Lehrkräfte zur Förderung des Transfers vor allem solche Aufgaben einsetzen, die neu sind, aber dennoch einen hohen Bezug zu ihrer Lebenswelt aufweisen. Zweitens können Schülerinnen und Schüler dazu angeregt werden, ihr neues Wissen zu reflektieren und eigene Beispiele und Anwendungen für das Gelernte zu entwickeln. Hierzu bieten sich vor allem Lerntagebücher an, in denen Schülerinnen und Schüler dazu aufgefordert werden, darüber nachzudenken, welches Verständnis sie über den neuen Lerninhalt erreicht haben, Bezüge zur eigenen Lebenswelt herzustellen und zu überlegen, wie sie mögliche Verständnisschwierigkeiten beseitigen können (Nückles et al. 2020). Lerntagebücher werden individuell erstellt, weshalb sie besonders für das Lernen im häuslichen Umfeld geeignet sind (Klieme 2020). Zugleich können Lerntagebücher dazu beitragen, dass Schülerinnen und Schüler durch den Einsatz von Lernstrategien, die sie beim Schreiben anwenden, ihre Selbstregulationsfähigkeiten verbessern (Fischer et al. 2020). Digital erstellte Lerntagebücher können Lehrkräfte auch über die Distanz einsehen und Rückmeldung zur Qualität des Lernstrategieeinsatzes geben.

### Wie kann Rückmeldung das Lernen von Schülerinnen und Schülern unterstützen?

In Zeiten einer Pandemie ist es möglich, dass Schülerinnen und Schüler über längere Phasen nicht wie üblich im Klassenkontext lernen müssen. Ohne direkte Hinweise von Lehrkräften und Vergleiche mit den Mitschülerinnen und Mitschülern fehlen den Schülerinnen und Schülern wichtige Informationen, um einzuschätzen, wo sie im Lernprozess stehen. Aus diesem Grund spielt die Rückmeldung als eine Möglichkeit der Lehrkraft, konstruktive Unterstützung unter den Einschränkungen einer Pandemie umzusetzen, eine wichtige Rolle. Allerdings gab ein Großteil der Eltern in Befragungen zum Lernen im häuslichen Umfeld während der coronabedingten Schulschließung an, dass ihre Kinder eher selten eine Rückmeldung von den Lehrkräften erhielten (Wildemann und Hosenfeld 2020; Wößmann et al. 2020). Gleichzeitig stellte das Geben von Rückmeldung an die Schülerinnen und Schüler eine der größten Belastungen für Lehrkräfte dar (Forsa Politik- und Sozialforschung 2020; Vodafone Stiftung Deutschland 2020a).

Feedback als jegliche Information eines Lernenden über Aspekte der eigenen Leistung oder des Verstehens (Hattie und Timperley 2007) dient zur Verringerung der Abweichung zwischen dem aktuellen Lernstand und dem intendierten Ziel des Lernens. Auf der Basis einer Forschungssynthese entwickelten Hattie und Timperley (2007) ein Modell zur Gestaltung effektiver Rückmeldungen. Nach diesem Modell sind Rückmeldungen dann effektiv, wenn sie Informationen zum Lernziel (*Feed Up*: Wo gehe ich hin?), zum aktuellen Lernstand (*Feed Back*: Wo stehe ich?) und zu den Strategien, um dem Lernziel näher zu kommen (*Feed Forward*: Wie komme ich dahin?) geben. Sie beziehen sich nicht nur auf das Ergebnis der Aufgabenbearbeitung, sondern auch auf motivationale, kognitive und metakognitive Lernprozesse von Schülerinnen und Schülern.

Welche Möglichkeiten haben Lehrkräfte, unter den Einschränkungen einer Pandemie effektive Rückmeldung zu geben? Lehrkräfte können (1) digitale Programme einsetzen, damit die Schülerinnen und Schüler eine effektive Rückmeldung über ihr Lernen erhalten, und (2) Arbeitsblätter, die in der Coronazeit häufig von den Schülerinnen und Schülern zu Hause bearbeitet wurden (z. B. Vodafone Stiftung Deutschland 2020a), so gestalten, dass Schülerinnen und Schüler über ihren Lernprozess informiert werden.

Obwohl nach der Umfrage von Forsa (2020) Lehrkräfte bislang selten digitale Diagnosewerkzeuge einsetzen, sind diese im Sinne des *Feed Back* geeignet, Schülerinnen und Schülern unmittelbar und individuell Rückmeldung auch über räumliche Distanz zu geben (McLaughlin und Yan 2017; Van der Kleij et al. 2015). Insbesondere für die Rückmeldung zum Üben der Schülerinnen und Schüler bieten sich digitale Werkzeuge an. Über diese Werkzeuge erhalten nicht nur Schülerinnen und Schüler unmittelbar Feedback, sondern auch die Lehrkräfte einen Überblick über den Lernstand ihrer Schülerinnen und Schüler. Elaboriertes Feedback, das nicht nur die Antwort auf die Korrektheit des Aufgabenergebnisses gibt, kann in digitalen Lernprogrammen durch zusätzliche Informationen umgesetzt werden, beispielsweise durch ergänzende Erklärungen, Lösungsansätze oder zusätzliche Hinweise zu der Regulation des Lernprozesses (z. B. Van der Kleij et al. 2015). So können Schülerinnen und Schüler auch unter Pandemiebedingungen im Sinne des *Feed Forward* Informationen darüber erhalten, wie sie den Lernzielen näherkommen können. Mit digitalen Diagnosewerkzeugen ist es zudem einfacher, die Verstehensprozesse der Schülerinnen und Schüler in kürzeren Abständen (z. B. einmal pro Woche) zu diagnostizieren. Dadurch kann lernprozessbegleitend ein noch systematischerer Wissensaufbau gefördert werden. Diese Art des formativen Assessment wird in Deutschland bisher selten eingesetzt, obwohl empirische Studien ihre Wirksamkeit belegen (z. B. Souvignier 2018).

Auch beim Lernen mit Arbeitsblättern lassen sich die Prinzipien effektiven Feedbacks realisieren. Hierzu können Lehrkräfte als Ergänzung zu den Arbeitsblättern Musterlösungen erstellen, die den Schülerinnen und Schülern nach dem Lernen zum Vergleich mit der eigenen Lösung zu Verfügung gestellt werden. Neben der Information über die Korrektheit des Aufgabenergebnisses sollten Lehrkräfte diese Musterlösungen mit Informationen über richtige Lösungsansätze und -wege anreichern und Hinweise geben, wie und an welcher Stelle die Überwachung des eigenen Lernprozesses umgesetzt werden sollte. Um den Prozess der Rückmeldung zu begleiten und zu strukturieren, können Leitfragen eingesetzt werden, die Schülerinnen und Schüler bei der Verwendung der Musterlösung unterstützen. Lehrkräfte mit einem hohen fachdidaktischen Wissen haben ein Bewusstsein für die typischen Fehlkonzepte und Hürden der Schülerinnen und Schüler bei der Beschäftigung mit dem Lernstoff (Krauss et al. 2008) und können Musterlösungen mit Leitfragen entwickeln.

### Welche Rolle spielen die Eltern für das Lernen ihrer Kinder und wie können Lehrkräfte die Eltern unterstützen?

Für die Eltern von Schülerinnen und Schülern besteht in Zeiten einer Pandemie die Herausforderung darin, in das Lernen ihrer Kinder im häuslichen Umfeld nicht zu viel einzugreifen und gleichzeitig soviel Unterstützung wie nötig zu geben (Pomerantz et al. 2006). Zur Förderung der Motivation der Schülerinnen und Schüler ist die Orientierung des elterlichen Verhaltens an den drei Grundbedürfnissen der Selbstbestimmungstheorie (d. h. Kompetenzerleben, Autonomieerleben, soziale Eingebundenheit) sinnvoll (Deci und Ryan 1985). So ein elterliches Verhalten zeichnet sich durch *Ansprechbarkeit*, *Autonomieunterstützung* und *Strukturierung* aus (Dumont et al. 2014). Eltern sollten demzufolge ansprechbar bei Problemen sein, jedoch nicht kontrollierend handeln, ganz im Sinne einer konstruktiven Unterstützung. Beispielsweise könnten Eltern aufmerksam gegenüber den Lösungsansätzen des Kindes sein, zur selbstständigen Bearbeitung von Aufgaben ermutigen, den Umgang mit Misserfolgen unterstützen und sensibel für Fortschritte des Kindes sein. Mindestens ebenso wichtig wie eine solche motivationsförderliche elterliche Unterstützung ist der Aspekt der Strukturierung durch die Eltern. Sie bezieht sich auf die lernförderliche Gestaltung des Lernumfeldes durch die Sicherstellung einer guten Arbeitsumgebung (z. B. Abschirmung von ablenkenden Reizen) sowie auf Regeln und Zeitstrukturen für das Lernen im häuslichen Umfeld. Ergebnisse aus der Hausaufgabenforschung zeigen, dass eine solche elterliche Unterstützung positiv mit dem Lernverhalten der Schülerinnen und Schüler assoziiert ist, wohingegen viel Kontrolle durch die Eltern sogar nachteilig wirken kann (Dumont et al. 2014).

Für die ausgedehnten Phasen des individuellen Lernens im häuslichen Umfeld unter Pandemiebedingungen dürfte die elterliche Unterstützungsqualität eine besondere Rolle spielen (Köller et al. 2020): Je länger die Phasen des individuellen Lernens im häuslichen Umfeld sind, umso höher sind auch die Anforderungen an die Schülerinnen und Schüler wie auch an deren Eltern. Eltern wendeten in der Coronazeit erwartungsgemäß mehr Zeit für die Betreuung des Lernens ihrer Kinder auf (Wildemann und Hosenfeld 2020). Ein Großteil der Eltern gab dennoch an, relativ gut mit der Situation umgehen zu können (Vodafone Stiftung Deutschland 2020b). Jedoch sahen drei Viertel aller befragten Eltern Schwierigkeiten darin, die Lernunterstützung zu Hause über einen längeren Zeitraum aufrechtzuerhalten. Zudem wurde relativ häufig von Streit zwischen Eltern und Kindern berichtet (Vodafone Stiftung Deutschland 2020b; Wößmann et al. 2020). Für ein Drittel der Eltern stellte die Schulschließung eine große psychische Belastung dar (Wößmann et al. 2020). Sich negativ verstärkende Prozesse in der lernbezogenen Interaktion zwischen Eltern und deren Kindern gilt es daher zu vermeiden. Studien aus der Hausaufgabenforschung zeigen, dass Eltern dazu tendieren, kontrollierend und mit Druck auf ungünstiges Lernverhalten ihrer Kinder zu reagieren, was wiederum ungünstiges Verhalten auf Seiten der Kinder verstärken und eine Negativspirale in Gang setzen kann (z. B. Moroni et al. 2016).

Vor diesem Hintergrund sollten Lehrkräfte bei der Förderung des Lernens ihrer Schülerinnen und Schüler unter Pandemiebedingungen auch die Elternseite berücksichtigen. Dies kann direkt durch Informationen an die Eltern über eine motivationsförderliche häusliche Unterstützungskultur erfolgen. Zudem können Lehrkräfte indirekt durch die sorgfältige Planung des Lernens im häuslichen Umfeld die Anforderungen an die Eltern reduzieren: Je weniger Fragen bei den Schülerinnen und Schülern offen bleiben, desto weniger ist elterliche Unterstützung notwendig. Daher ist eine sorgfältige Planung auch ein wichtiger Hebel, um zu verhindern, dass die Leistungsschere zwischen Schülerinnen und Schülern unter Pandemiebedingungen weiter auseinander geht. Ergebnisse einer Befragung von über 1000 Eltern deuten auf eine solche Weitung der Leistungsschere hin (Wößmann et al. 2020). Leistungsschwächere Schülerinnen und Schüler reduzierten nicht nur deutlich stärker als leistungsstärkere Schülerinnen ihre schulischen Aktivitäten, sondern erhöhten auch gleichzeitig ihren Anteil an passiven Tätigkeiten wie Handy-Nutzung oder Fernsehen. Zudem fühlten sich vor allem Eltern mit formal niedrigem Bildungsniveau stark belastet und befürchteten, dass ihr Kind den Anschluss an den Schulstoff verliert (Vodafone Stiftung Deutschland 2020b).

## Schlussfolgerungen und Diskussion

In diesem Artikel haben wir dargestellt, wie man Ergebnisse aus Unterrichts- und Instruktionsforschung nutzen kann, um Hinweise abzuleiten, wie Unterrichten auch unter den Einschränkungen einer Pandemie gelingen kann. Wir haben aufgezeigt, dass für eine Diskussion über guten Unterricht unter Pandemiebedingungen eine ausschließliche Betrachtung auf der Ebene von Sichtstrukturen des Unterrichts nicht zielführend ist. Stattdessen ist es aufschlussreicher, die Basisdimensionen der Unterrichtsqualität, d. h. Potenzial zur kognitiven Aktivierung, konstruktive Unterstützung und Effizienz des Klassenmanagements, heranzuziehen, um das Unterrichten in Zeiten einer Pandemie zu analysieren. Um die Frage zu beantworten, wie guter Unterricht im Sinne der Basisdimensionen unter Pandemiebedingungen zu gestalten ist, haben wir Erkenntnisse aus der Instruktionsforschung präsentiert, aus denen Empfehlungen für die Gestaltung zentraler Lehr-Lern-Elemente ableitbar sind.

### Empfehlungen

Zusammenfassend lassen sich folgende Empfehlungen geben:

- Um ihrer Verantwortung für das Lernen der Schülerinnen und Schüler im häuslichen Umfeld nachzukommen, sollten Lehrkräfte ihren Unterricht auf der Grundlage von Lernzielen systematisch planen. Durch die Analyse der Lernziele können Lehrkräfte feststellen, welches Wissen Schülerinnen und Schüler im Unterricht erwerben müssen. Diese Informationen sind wichtig, um den Lernprozess für Schülerinnen und Schüler so zu gestalten, dass Unklarheiten, auf die Lehrkräfte nicht unmittelbar reagieren können, möglichst reduziert werden. Zudem können zu hohe Anforderungen an die Selbstregulationsfähigkeiten der Schülerinnen und Schüler durch gut strukturierte Lernmaterialien vermieden werden.
- Besonders beim Lernen allein im häuslichen Umfeld besteht die Gefahr, dass Schülerinnen und Schüler unmotiviert sind. Um dieser Gefahr vorzubeugen, sollten den Schülerinnen und Schülern Informationen über die Lernziele gegeben werden. Zudem sind Aufgaben mit einem direkten Bezug zur eigenen Lebenswelt hilfreich, um den Nutzen eines Lerninhaltes zu verdeutlichen. Auch die Förderung realistischer Erwartungen durch Informationen über das bevorstehende Lernen kann die Motivation für die Auseinandersetzung mit den Lerninhalten fördern.
- Eine produktive Auseinandersetzung mit neuen Lerninhalten in Erklärungen kann scheitern, wenn bei Schülerinnen und Schülern Verständnisschwierigkeiten entstehen, die sie ohne Unterstützung der Lehrkraft nicht selbständig klären können. Deshalb sollten Erklärungen die Anwendung des zu erwerbenden Wissens anhand von Beispielen veranschaulichen. Dabei empfiehlt es sich, mehrere Beispiele zu präsentieren, um den Aufbau eines flexiblen Wissens zu fördern. Zur Steigerung der Lernwirksamkeit von Erklärungen kann eine multimediale Aufbereitung nützlich sein, wenn sie lernpsychologische Gestaltungsprinzipien berücksichtigt. Bei der Nutzung von Erklärvideos sollte vor allem darauf geachtet werden, dass Schülerinnen und Schüler die dargestellten Informationen ihrem individuellen Lernprozess entsprechend verarbeiten können. Zudem kann es nützlich sein, dass Lehrkräfte für das Lernen aus Erklärvideos notwendige Strategien vermitteln.
- Wenn Schülerinnen und Schüler nicht wie üblich von ihrer Lehrkraft im Lernprozess unmittelbar begleitet werden können, müssen sie selbst eine aktive Rolle für ihr Lernen übernehmen. Ansonsten besteht das Risiko, dass sie ihr Lernen zu früh aufgeben. Deshalb ist es wichtig, dass Schülerinnen und Schüler nach der erstmaligen Auseinandersetzung mit neuen Lerninhalten weitere Lernaktivitäten durchführen. Dadurch werden kognitive und metakognitive Prozesse angeregt, die ihren Wissenserwerb unterstützen. Lehrkräfte sollten bei Schülerinnen und Schülern diese Lernaktivitäten ausdrücklich anstoßen und ihnen Rückmeldung zur Qualität ihrer Durchführung geben.
- Das Üben ist ein zentraler Bestandteil des Lernens und erfolgt auch im herkömmlichen Unterricht häufig individuell. Besonders beim Lernen über Distanz, auf das Lehrkräfte nicht wie gewohnt direkt Einfluss nehmen können, sollte das Potenzial des Übens für das Lernen ausgeschöpft werden. Hierzu empfiehlt es sich, Übungsaufgaben zu verwenden, die auf die zu erreichenden Lernziele abgestimmt sind und von Schülerinnen und Schülern eine aktive Anwendung ihres erworbenen Wissens erfordern. Zudem kann das Lernen unterstützt werden, wenn Lehrkräfte vorgeben, wie Schülerinnen und Schüler die Übungsaufgaben zeitlich verteilt bearbeiten sollen. Schließlich kann durch die Reihenfolge der zu bearbeiten Übungsaufgaben verhindert werden, dass Schülerinnen und Schüler nicht verstehen, in welchen Situationen sie ihr erworbenes Wissen anwenden können.
- Die möglichen Einschränkungen einer Pandemie können dazu führen, dass im Mittelpunkt des Unterrichtens das Wiederholen von Lerninhalten steht. Um ein vertieftes Verständnis von Lerninhalten im Sinne eines erfolgreichen Transfers zu unterstützen, sollten Lehrkräfte Schülerinnen und Schüler ihr Wissen auch in authentischen Lebenssituationen anwenden lassen. Hierzu können Lehrkräfte Aufgaben vorgeben oder bei Schülerinnen und Schülern eine Reflexion über die Übertragbarkeit ihres Wissens auf persönliche relevante Lebenssituationen anregen.
- Beim Lernen allein im häuslichen Umfeld fehlen die übliche direkte Rückmeldung durch die Lehrkräfte sowie die Vergleiche mit den Mitschülerinnen und Mitschülern. Es ist daher besonders wichtig, dass Schülerinnen und Schüler gezielt Informationen von den Lehrkräften über ihr Lernen erhalten. Eine solches Feedback kann mit digitalen Werkzeugen oder über Materialien wie strukturierte Musterlösungen, die den Schülerinnen und Schülern nach dem Lernen zum Vergleich mit den eigenen Lösungen bereitstehen, umgesetzt werden.
- Um zu vermeiden, dass Schülerinnen und Schülern aus Familien mit geringeren Unterstützungsmöglichkeiten Nachteile entstehen, sollten Lehrkräfte dazu beitragen, beim individuellen Lernen im häuslichen Umfeld die Anforderungen an die Eltern zu reduzieren. Dies können Lehrkräfte durch gut strukturierte Lernmaterialien erreichen, die wenig Elternunterstützung erfordern. Zudem sollten Lehrkräfte die Eltern über das Lernen der Kinder informieren und konkrete Hinweise geben, wie eine motivationsförderliche häusliche Unterstützungskultur erreicht werden kann.
- Lehrkräfte sollten digitale Werkzeuge didaktisch sinnvoll einsetzen, um das Lernen der Schülerinnen und Schüler zu fördern. Digitale Werkzeuge können von Lehrkräften genutzt werden, um Erklärungen multimedial zu gestalten. Aber auch Schülerinnen und Schüler können digitale Werkzeuge einsetzen, um sich mit neuen Lerninhalten vertieft auseinanderzusetzen (z. B. Visualisierungen auf Tablet erstellen), Übungsaufgaben zu bearbeiten (z. B. Online-Testaufgaben bearbeiten) oder über das Lernen zu reflektieren (z. B. digitales Lerntagebuch schreiben). Die erstellten Produkte können Lehrkräfte einsehen, um Schülerinnen und Schülern Rückmeldung über ihr Lernen zu geben.

Es ist zu berücksichtigen, dass die von uns dargestellten Empfehlungen keine direkten Handlungsanweisungen für Lehrkräfte darstellen. Sie können aber wichtige Anhaltspunkte liefern, welche Aspekte eine Lehrkraft bei der Planung des Unterrichts beachten sollte, um das Lernen ihrer Schülerinnen und Schüler unter Pandemiebedingungen zu unterstützen. Wie die einzelnen Lehr-Lern-Elemente konkret zu gestalten sind, ist unter Berücksichtigung der Besonderheiten des jeweiligen Fachs und durch weitere didaktische Überlegungen zu entscheiden. Allein bei der Gestaltung einer Erklärung müssen viele didaktische Entscheidungen getroffen werden (Koedinger et al. 2013): Wie viele Beispiele sollten in der Erklärung präsentiert werden? Wie konkret sollten die Beispiele gestaltet sein? Wie sollen die Schülerinnen und Schüler die Informationen in der Erklärung weiterverarbeiten? Wie sollten Übungsaufgaben zur Anwendung des Wissens zeitlich gestaltet sein? Wann sollte Rückmeldung zum Üben gegeben werden?

Zudem ist darauf hinzuweisen, dass die Empfehlungen, die wir geben, eine zusammenfassende Beschreibung von Erkenntnissen aus der Forschung darstellen. Für jedes Lehr-Lern-Element liegen in der Regel differenziertere Erkenntnisse vor, die für die erfolgreiche Umsetzung von Unterricht unter Pandemiebedingungen wichtig sein können, die wir aber nicht im Detail behandeln konnten. Als ein Beispiel seien die Richtlinien für die Gestaltung von Lösungsbeispielen (Renkl 2014) genannt.

Obwohl wir in unseren Empfehlungen nicht gesondert auf Unterschiede zwischen Schülerinnen und Schülern eingehen, sind die individuellen Lernvoraussetzungen der Schülerinnen und Schüler beim Unterrichten zu berücksichtigen. Beispielsweise ist bekannt, dass die Lernwirksamkeit von Lösungsbeispielen mit zunehmendem Vorwissen von Lernenden sinkt (Renkl 2014). Auch wird in Bezug auf die Reihenfolge von Übungsaufgaben empfohlen, lernschwächeren Schülerinnen und Schülern nacheinander mehrere Aufgaben zur Festigung derselben Fertigkeiten zu geben (Hughes und Lee 2019).

Weiterhin rücken wir in unseren Empfehlungen den Unterricht und die Möglichkeiten der Lehrkräfte, das Lernen unter Pandemiebedingungen zu unterstützen, in den Mittelpunkt. Ein weiterer wichtiger Ansatzpunkt ist die Förderung des selbstregulierten Lernens der Schülerinnen und Schüler (Fischer et al. 2020). Auch Trainings für Eltern, um motivationsunterstützendes Verhalten gegenüber ihren Kindern zu fördern und Streit zu reduzieren, werden vorgeschlagen (Köller et al. 2020).

Schließlich ist zu berücksichtigen, dass die Erkenntnisse, die wir in den Empfehlungen präsentieren, häufig aus experimentellen Laborstudien stammen, in denen die Lernwirksamkeit von Merkmalen einzelner Lehr-Lern-Elemente isoliert untersucht wird. Daher wäre es im Sinne einer hohen ökologischen Validität wünschenswert, in zukünftigen Studien das Lernen unter den natürlichen Bedingungen einer Pandemie zu untersuchen.

### Zukünftige Forschung

Die Befragungen zum Lernen in Zeiten von Corona (z. B. Forsa Politik- und Sozialforschung 2020; Huber et al. 2020; Vodafone Stiftung Deutschland 2020a, 2020b; Wildemann und Hosenfeld 2020; Wößmann et al. 2020) zeigen, wie Lehrkräfte das Unterrichten unter den Einschränkungen von Corona umsetzten und wie Schülerinnen und Schüler in dieser besonderen Situation lernten. Allerdings wird in den Befragungen nicht systematisch die Qualität des Unterrichtens aus einer pädagogisch-psychologischen Perspektive untersucht.

Vor diesem Hintergrund wäre es wünschenswert, das Unterrichten unter Pandemiebedingungen nicht nur im Hinblick auf die Basisdimensionen der Unterrichtsqualität (Praetorius et al. 2018) zu analysieren, wie wir es in diesem Beitrag getan haben, sondern auch empirisch zu untersuchen. Es wären Studien aufschlussreich, die sich mit der Frage beschäftigen, welche Merkmale der Unterrichtsqualität für das Lernen unter den Einschränkungen einer Pandemie besonders wichtig sind.

Auch könnten längsschnittliche Studien mit repräsentativen Daten über die Kompetenzentwicklung von Schülerinnen und Schülern zur Beantwortung der gesellschaftlich wichtigen Frage beitragen, inwieweit eine Vergrößerung sozialer Ungleichheit zwischen Schülerinnen und Schülern unter Pandemiebedingungen auftritt. Dabei könnte auch die Rolle individueller, familiärer und institutioneller Merkmale für eine mögliche Vergrößerung sozialer Ungleichheit analysiert werden (Baumert et al. 2003).

Zudem sollte in experimentellen Studien untersucht werden, unter welchen Bedingungen Merkmale zur Gestaltung des Unterrichtens unter Pandemiebedingungen (z. B. Beispiele in Erklärungen) – auch unter Einsatz digitaler Werkzeuge – das Lernen besonders fördern. Dieser Frage könnte zusätzlich in Abhängigkeit der individuellen Lernvoraussetzungen von Schülerinnen und Schülern nachgegangen werden. Beispielsweise wären Erkenntnisse darüber interessant, welche individuellen Lernvoraussetzungen (z. B. Selbstregulationsfähigkeiten, Vorwissen) Schülerinnen und Schüler mitbringen müssten, um von den langen Phasen des individuellen Lernens zu profitieren, und wie Schülerinnen und Schüler mit Schwierigkeiten beim individuellen Lernen über längere Phasen optimal unterstützt werden können. Daraus wären Implikationen in Hinblick auf die Individualisierung von Unterrichtsangeboten ableitbar, die auch für die zukünftige schulische Praxis von Bedeutung sind.

Schließlich könnte sich zukünftige Forschung der Frage widmen, wie unter Pandemiebedingungen die längeren Phasen des individuellen Lernens, in denen digitale Medien zum Einsatz kommen, mit Präsenzphasen im herkömmlichen Unterricht kombiniert werden sollten. Ein bekannter Ansatz ist *flipping the classroom* (DeLozier und Rhodes 2017), der darin besteht, sich mit einem Lerninhalt unter Verwendung digitaler Medien individuell auseinanderzusetzen (z. B. in Form von Erklärvideos), um anschließend das erworbene Wissen in Präsenzphasen gemeinsam anzuwenden (z. B. Aufgaben bearbeiten). Die Forschung legt nahe, dass der Ansatz zu einem hohen Lernerfolg führen kann, wenn die Präsenzphase nicht verkürzt wird und das Wissen von Lernenden überprüft wird (z. B. van Alten et al. 2019). Allerdings wurde *flipping the classroom* bislang vor allem im Hochschulbereich untersucht. Deshalb sollten sich zukünftige Studien noch stärker mit der Übertragbarkeit des Ansatzes auf das Lernen von Schülerinnen und Schülern beschäftigen und auch Hinweise liefern, wie der Ansatz möglichst lernwirksam zu gestalten ist.

Zusammenfassend sind wir überzeugt, dass sich die durch Corona entstandene Ausnahmesituation langfristig als Chance für das Bildungssystem begreifen lässt: Regt die Ausnahmesituation vermehrt Forschung zum Unterrichten und Lernen unter Pandemiebedingungen an, können lernwirksame und innovative Lernmaterialien und Lernsettings für individuelle Lernphasen entstehen, die in besonderem Maße den individuellen Bedürfnissen in heterogenen Schülerinnen- und Schülergruppen gerecht werden könnten. Wirksame digitale Lerntools sowie strukturierte analoge Formen des individuellen Lernens außerhalb des Klassenverbandes könnten so dauerhaft den regulären Präsenzunterricht anreichern.
